# True Impact of Sex Differences in Diagnosis and Management of Cardiovascular Disease

**DOI:** 10.7759/cureus.99237

**Published:** 2025-12-14

**Authors:** Lana Alamat, Aakash Rana, Angel Lopez Candales, Khalid Sawalha, Allison Shaw-Devine

**Affiliations:** 1 Internal Medicine, Jordan Hospital, Amman, JOR; 2 Medicine, Central Arkansas Veterans Healthcare System, Little Rock, USA; 3 Cardiovascular Medicine, University of Puerto Rico School of Medicine, San Juan, USA; 4 Cardiovascular Disease, University of Arkansas for Medical Sciences, Little Rock , USA; 5 Cardiometabolic Medicine, University of Missouri Kansas City School of Medicine, Kansas City, USA; 6 Cardiovascular Disease, University of Arkansas for Medical Sciences, Little Rock, USA

**Keywords:** cardiovascular, coronary artery disease, differences, gender, sex

## Abstract

Over the years, scientific advancements have enhanced our understanding of cardiovascular disease (CVD) in adults. Unfortunately, progress has been less substantial in understanding the manifestations of CVD in women. In light of this discrepancy, the goal of this review is to outline specific differences between men and women that appear to place women at increased risk of poorer outcomes from cardiovascular disease compared to their male counterparts. A better understanding of these differences may provide clinicians with an advantage in recognizing potential areas to target for improving outcomes among women. By adapting strategies for both prevention and treatment in this large population, we may help mitigate the longstanding disparities that persist.

## Introduction and background

Over the past few decades, considerable effort has been made to improve the medical community’s understanding of sex-based differences in outcomes and treatment strategies for managing women with cardiovascular disease. The American Heart Association (AHA) has led the charge in advocating for higher-quality cardiovascular care for women. In 1997, the AHA launched a campaign to raise awareness of women's risk for developing cardiovascular disease and the differences in presentation compared to men [1]. Since then, death rates from CVD among women have decreased by nearly half [1-3]. Despite these efforts, CVD remains the leading cause of death in women, accounting for nearly one out of every three deaths annually in the United States [4]. Consequently, the intent of this review is not only to highlight how sex differences place women at greater risk for adverse cardiovascular outcomes but also to emphasize strategies for improved recognition, preventive measures, and timely implementation of effective diagnostic and treatment options to minimize disparities in clinical care.

*“For it all depends on how we look at things, and not on how they are in themselves”* - C.G. Jung. This quote by the Swiss psychiatrist and founder of analytical psychology aptly introduces the discussion on gender differences in CVD. A comprehensive approach to women's health must include CVD risk assessment. Historically, much of the focus has rightly been on breast cancer diagnosis and treatment among middle-aged women [5]. Similarly, pregnancy-related diabetes and hypertension (HTN) have received attention as important areas of women's health research and international grant funding [6,7]. However, we remain far from closing the gaps in knowledge and care delivery needed to reduce sex-based disparities in cardiovascular outcomes [6].

Two essential elements contribute to the development of CVD in women: (1) sex, which predisposes women through sex-specific gene expression; and (2) gender, which affects women through environmental factors and epigenetic modifiers [5]. Unfortunately, many healthcare providers remain unaware of these differences, potentially delaying diagnosis and care due to symptom variability and test interpretation challenges, such as increased false positives in women due to differences in coronary artery diameter, hormonal influences, and left ventricular size. The management of CVD should not follow a 'one-size-fits-all' model. A tailored, inclusive approach is necessary to address disparities and improve outcomes for women.

This review aims to raise awareness of outcome differences between men and women under current treatment paradigms and to advocate for more inclusive methods of prevention and treatment to continue reducing cardiovascular mortality among women. We organize this review by major CVD entities, such as ischemic heart disease (IHD), heart failure (HF), HTN, aortic valve disease (AVD), stroke and carotid stenosis, peripheral arterial disease, and diabetes. 

## Review

Ischemic heart disease

To begin, although women as a group are less likely to develop obstructive coronary artery disease (CAD), they more commonly experience more myocardial ischemia than their male counterparts. As such, the term 'ischemic heart disease' (IHD) may be more appropriate than CAD when referring to women [8]. For example, ischemia with no obstructive coronary arteries (INOCA) and myocardial infarction with no obstructive coronary arteries (MINOCA) are more common in women than men. Women also tend to have worse outcomes with these conditions, including a higher risk of future cardiac events, stroke, and a reduced quality of life compared to men with the same conditions [9]. These conditions often involve microvascular issues, and the higher prevalence in women in Western societies [9].

Among women aged 45 to 65, distinct abnormalities in the coronary microvasculature are considered the most likely cause of IHD. Additionally, type 1 diabetes, gestational diabetes, or preeclampsia increases the risk of CAD in younger women [10-14]. Later in life, type 2 diabetes significantly contributes to the burden of CAD in women [15]. Moreover, cardiometabolic risk factors, including prediabetes, inflammation markers, and endothelial dysfunction, further elevate women’s cardiovascular risk [16-19].

While acute coronary syndrome (ACS) is more common in men under 60, women with non-ST-elevation myocardial infarction (NSTEMI) often demonstrate myocardial ischemia without coronary stenosis [9-10]. Accordingly, microvascular disease, coronary artery spasm, and spontaneous coronary artery dissection should be considered in women [20,21]. Myocardial infarction with non-obstructive coronary arteries refers to myocardial infarction occurring without significant coronary obstruction, defined as no lesions exceeding 50% stenosis [20,21]. In contrast, INOCA describes anginal symptoms with angiographic stenosis below 50% [20,21]. A persistent diagnostic gap exists, as many clinicians stop after a 'normal' angiogram, resulting in systematic under-evaluation of women with ischemic symptoms and contributing to missed or delayed diagnosis of MINOCA and INOCA.

Regarding the use of coronary angiography post-ACS, women have a lower adjusted odds ratio (OR) for undergoing percutaneous coronary intervention (PCI) than men (OR 0.95, 95% CI 0.92-0.99) and are less frequently referred for coronary artery bypass grafting (CABG) (OR 0.81, 95% CI 0.76-0.87). Women with single-vessel disease are less likely to receive PCI compared to men, whereas women with multi-vessel disease are more likely to receive intervention compared to men. Women also experience more in-hospital complications post-PCI [22].

Importantly, women still face delays in receiving medical care for ACS symptoms [23]. Although troponin levels tend to be lower in women, high-sensitivity assays with sex-specific cutoffs have improved MI diagnosis accuracy [24,25].

Heart failure and cardiomyopathies

By 2030, HF prevalence is projected to exceed 8 million cases in the US, with direct costs rising from $21 billion in 2012 to $53 billion [26]. Approximately half of HF patients have preserved ejection fraction (HFpEF) [26-27]. This growing epidemic is likely linked to rising rates of HTN, obesity, and metabolic syndrome [28].

Current data indicate that HFpEF disproportionately affects women, with a female-to-male ratio of 2:1 [29-30]. Interestingly, males with HFpEF tend to experience worse clinical outcomes, such as higher risks of HF hospitalization, cardiovascular death, and composite HF events, compared with women [31,32]. While men are more prone to right ventricular failure and sudden cardiac death, women more frequently die from non-cardiovascular causes [33-35]. The PARAGON-HF (Prospective Comparison of ARNI with ARB Global Outcomes in Heart Failure with Preserved Ejection Fraction) trial found that sacubitril-valsartan was more effective in reducing HF hospitalizations in women than in men with HFpEF [36].

Similarly, in the TOPCAT (Treatment of Preserved Cardiac Function Heart Failure With an Aldosterone Antagonist) trial, spironolactone appeared to have a sex-specific effect: women showed benefit across the full range of ejection fractions, whereas men derived benefit only at lower ejection fractions [37]. However, substantial regional heterogeneity within TOPCAT (e.g., Russia/Georgia vs. the Americas) adds complexity and makes the overall treatment effect more difficult to interpret.

In the context of heart failure with reduced ejection fraction (HFrEF), women differ markedly from men in terms of clinical profile, response to therapy, and long-term outcomes. A combined analysis of the PARADIGM-HF (Prospective comparison of Angiotensin Receptor-neprilysin inhibitor (ARNI) with Angiotensin-converting enzyme inhibitor (ACEI)) and ATMOSPHERE (Aliskiren Trial of Minimizing Outcomes in Patients With Heart Failure) trials involving 12,058 men and 3,357 women with HFrEF found that although women live longer, they report worse quality of life and greater psychological and physical disability [38]. Furthermore, women with HFrEF appear to benefit more from cardiac resynchronization therapy and less from digoxin [39,40]. 

Several evidence-supported explanations exist to support such differences. Lead-time bias may contribute, as women are often diagnosed at earlier HF stages, which can artificially enhance observed survival [39]. Women also have lower non-cardiovascular competing mortality, which can preserve overall survival even with high HF symptom burden [38]. In addition, women consistently report worse symptoms across CVDs, raising the possibility that sex-based differences in symptom perception or incomplete sex validation of quality-of-life instruments influence reported outcomes [40]. True biological differences have also been described: women exhibit more favorable ventricular-arterial coupling, slower HF progression, and heightened natriuretic peptide signaling at equivalent hemodynamic stress, which may support survival despite worse functional status [37-40]. Quality-of-life measures should therefore be interpreted with caution, as their performance may differ by sex.

Despite these insights, sex-specific experiences in HFrEF are poorly characterized, and the clinical community has yet to address these disparities fully. Regarding cardiomyopathies, both dilated and hypertrophic forms, despite equal genetic distribution, are more frequently diagnosed in men [41-43]. In heart transplantation, men are more often recipients, while women are more often donors [43].

Hypertension

Recent data using 24-hour ambulatory blood pressure monitoring reveal that men have higher blood pressure than age-matched premenopausal women [44]. This may explain men’s higher CVD and renal disease risk. After menopause, however, women’s blood pressure rises, and by ages 60 to 69, non-Hispanic Black and Hispanic women show higher readings than their male counterparts [45].

While HTN is most prevalent in older adults, up to 20% of young adults are hypertensive [46]. Research by Everett and Zajacova demonstrated that women in their 20s are less likely to have HTN than men but are 35% more likely to be aware of their condition, possibly due to differences in healthcare utilization [47,48].

Although the precise immunological mechanisms are not fully defined, T-cell-mediated pathways may contribute to sex differences in HTN [48]. Women exhibit a more anti-inflammatory immune profile, which may attenuate blood pressure elevation compared with the more pro-inflammatory profile observed in men [49]. Enhanced activity of the angiotensin II type-2 (AT2) receptor in women further promotes anti-inflammatory signaling and vascular protection [50]. These mechanisms may partly account for observed sex differences in blood pressure trajectories and differential responses to renin-angiotensin-aldosterone system (RAAS)-targeting therapies.

Beyond menopause, conditions such as polycystic ovary syndrome, particularly the hyperandrogenic phenotype, raise HTN risk [50]. Sex-based pharmacokinetic differences also affect antihypertensive drug responses: women generally have more body fat, different plasma protein levels, enzyme activity variations, and lower renal clearance, all contributing to drug bioavailability [51]. Lipid-soluble drugs tend to persist longer in women, while water-soluble drugs have greater exposure, and renal clearance is higher in men [51]. These factors may explain why women are more prone to adverse drug reactions (50% to 70%) and are more likely to be hospitalized for such events (60%) [52].

Aortic valve stenosis 

Aortic stenosis (AS) is the most common valvular heart disease in developed nations, with rising prevalence due to aging populations [53,54]. Key risk factors for AS are male sex, age, smoking, HTN, low-density lipoprotein cholesterol (LDL-C), and lipoprotein (a) (Lp(a)) [53-54]. Aortic valve disease represents the majority of valvular cases, with AS accounting for 47% and aortic regurgitation for 18% [55,56].

Non-rheumatic calcific AVD, the most common valvular disorder in high-income countries, affects more than 12 million individuals worldwide. Although male sex is a recognized risk factor, partly due to higher bicuspid valve prevalence, a cardiovascular health study (n=5,201, ≥65 years) reported a 2% incidence of AS, rising to 2.6% in those over 75, with men demonstrating both higher incidence and heavier valve calcification than women [54]. Independent risk factors include older age (≈2-fold increase per decade), male sex (≈2-fold excess risk), smoking (≈35% increased risk), HTN (≈20% increased risk), and elevated LDL-C and Lp(a). Women, however, typically present with a distinct phenotype characterized by a smaller annulus, greater valvular fibrosis relative to calcification, and more concentric LV remodeling. These differences influence procedural outcomes: women have higher rates of vascular complications but often better survival after transcatheter aortic valve replacement (TAVR), whereas men exhibit more calcific burden and paravalvular leak risk [57]. Incorporating these sex-specific phenotypes provides a more coherent link to the broader theme of sex differences across CVD.

Stroke and carotid stenosis

Every year, more than 795,000 people in the US experience a stroke, with approximately 610,000 of these being new-onset strokes [58]. Sex differences have been observed in stroke types, with women more likely to experience cardioembolic strokes and men more likely to have lacunar strokes [59]. The Framingham Heart Study, which aimed to assess sex differences in stroke incidence and post-stroke disability, found that women experienced their first-ever stroke at a significantly older age compared to men (75.1 years vs. 71.1 years). Women had a higher stroke incidence above the age of 85 and a lower incidence at all other ages, as well as a higher lifetime risk of stroke across all ages. They also exhibited significantly more disability in the acute phase and during the three- to six-month follow-up period [60].

In terms of carotid stenosis, men are more likely to develop the condition, but the risk of stroke is higher in women [59]. Additionally, after undergoing carotid endarterectomy, men were found to have a lower risk of perioperative stroke and mortality, indicating a protective effect [61]. This sex difference in carotid repair outcomes may be attributed to a complex interplay of hormonal differences, anatomical factors (such as smaller vessel diameters in women), the development of different plaque patterns in women, and other biological factors [62].

Women also have a higher prevalence of atrial fibrillation at older ages, which likely contributes to their greater burden of cardioembolic stroke. In population cohorts, AF prevalence rises steeply in women after age 75, narrowing or surpassing that of men and aligning with the observed shift toward embolic mechanisms in late-life stroke. Regarding disability, analyses from the Framingham cohort indicate that the excess functional impairment in women persisted even after adjustment for age and major comorbidities, suggesting sex-specific vulnerability rather than confounding by older age at stroke onset.

Aortic disease

Like other CVDs, aortic diseases, specifically abdominal aortic aneurysms (AAA), share similar risk factors, including smoking, advanced age, hypertension, hyperlipidemia, and male sex as major contributors. The prevalence of AAA is reported to be four to six times higher in men than in women [62,63]. However, despite these sex differences in development and progression, women not only have a higher risk of rupture but also experience greater morbidity after surgical procedures [62]. Postmenopausal women are at greater risk for AAA development, indicating that female hormones play a protective role against AAA [64]. Regarding thoracic aortic aneurysms and acute dissections, men are primarily affected, although to a lesser extent compared to AAA. Approximately 65% of individuals presenting for surgical repair due to acute dissection or thoracic aneurysm are men [65,66].

Current guideline practices also reflect sex differences in AAA recognition. Major societies recommend one-time ultrasound screening for men aged ≥65 with a smoking history, whereas routine screening is not recommended for women despite their higher rupture-related mortality, contributing to systematic under-diagnosis. Importantly, women experience rupture at smaller aortic diameters than men, a finding linked to smaller baseline aortic size and less aneurysmal dilatation before failure. These anatomical differences support consideration of sex-specific repair thresholds and more selective screening strategies for high-risk women.

Diabetes mellitus

Although the precise mechanisms explaining the sex differences in diabetes mellitus (DM) and its associated cardiac complications remain unclear, there is compelling evidence that DM is more prevalent in women than in men [67]. Women with diabetes have a threefold to sevenfold increased risk of developing CVD, compared to a threefold risk in men with diabetes [68]. A meta-analysis of 37 prospective cohort studies by Huxley et al. demonstrated that women with diabetes had a 50% higher risk of fatal CVD compared to diabetic men [69]. This difference may be explained by a cardiometabolic pathway, where obesity is more common in women, leading to a higher prevalence of cardiovascular complications [70]. Other potential sex-related contributors to higher mortality in women include smaller coronary vessel size, greater inflammatory involvement, and less favorable outcomes in diabetes treatment [71].

Despite advances in diagnosis and management, CVD remains the leading cause of death in the United States and worldwide [72]. Recent data by Roth et al. indicate that the number of CVD-related deaths continues to rise, accounting for one-third of all global deaths in 2019 [73]. Even within the U.S., there are persistent sex disparities in controlling HTN, diabetes, and dyslipidemia, as revealed by data from the 2001-2016 US National Health and Nutrition Examination Survey [74].

Global burden and risk factor control

In the 1990s, underrepresentation of women in clinical trials was well documented [75]. Despite an increase in the recognition of sex differences in CVD, this trend has not significantly improved over the past decade [76,77]. Traditionally, the exclusion of women was attributed to perceived biological variability due to hormonal fluctuations [78]. However, emerging evidence now implicates sex-specific gene transcription, reduced selection efficiency, and the accumulation of deleterious mutations in modulating disease prevalence [79].

The European Gender Medicine Network (EUGenMed) Cardiovascular Clinical Study Group has proposed a novel framework advocating the combined use of sex and gender to assess CVD risks [80]. According to this model, sex differences can often be validated through animal studies, whereas gender encompasses uniquely human sociocultural interactions, behaviors, relationships, and lifestyle factors, each of which influences cardiovascular health [81]. Despite ongoing confusion between the terms 'sex' and 'gender,' the aim is to properly frame them within a medical context with sincere regard for women's health.

Peripheral artery disease

Peripheral arterial disease (PAD) is underdiagnosed and undertreated in women despite being a significant marker of systemic atherosclerosis. In a 2010 US census evaluation, more women aged ≥40 years had PAD than men [82]. Women with PAD are more likely to present with atypical symptoms, such as fatigue or leg discomfort, rather than classic intermittent claudication, leading to under-recognition [83]. Moreover, atypical symptom presentation in women is frequently misinterpreted as musculoskeletal or neurologic conditions such as arthritis, peripheral neuropathy, or spinal stenosis due to overlapping clinical features and diagnostic ambiguity [84-85].

Ultimately, delayed diagnosis in women, due to either absent or predominantly atypical symptoms, has been associated with more advanced disease at presentation, including a higher incidence of chronic limb-threatening ischemia compared to men [84,85]. The ankle-brachial index (ABI) is the primary diagnostic tool for PAD. A single study demonstrated that men had worse specificities in both ABI and toe-brachial index, yet even the authors stated that this finding "should be taken with precaution since multivariate analysis did not show any association regarding diagnostic accuracy of ABI between men and women, and male gender is overrepresented in the meta-analysis" [86].

Noninvasive imaging consisting of duplex ultrasound, CT angiography, and magnetic resonance angiography has shown no sex- or gender-based differences in imaging accuracy for PAD [87]. The most significant barrier in diagnosing PAD in women likely remains clinician bias and a failure to recognize atypical symptoms [87]. Treatment disparities also exist: women are less likely to be referred for exercise therapy, vascular imaging, or revascularization procedures [88]. Women with PAD are often less likely to be treated with any lipid-lowering agent, as well as achieve target LDL-C [89]. Anti-thrombotic therapies are used less in women compared to men after revascularization [90]. Table 1 outlines key concepts we consider essential in evaluating the impact on women's cardiovascular health.

**Table 1:** Major key points in sex/gender differences in different CVDs CVDs: Cardiovascular diseases, IHD: Ischemic heart disease, HF: Heart failure, HTN: Hypertension, AS: Aortic stenosis, DM: Diabetes mellitus, PAD: Peripheral artery Disease, CAD: Coronary artery disease, PCI: Percutaneous coronary intervention, CABG: Coronary artery bypass grafting, HFpEF: Heart failure with preserved ejection fraction, RV: Right ventricle, SCD: Sudden cardiac death, ARNI: Angiotensin receptor–neprilysin inhibitor, HFrEF: Heart failure with reduced ejection fraction, QoL: Quality of life, CRT: Cardiac resynchronization therapy, BP: Blood pressure, ACS: Acute coronary syndrome, AAA: Abdominal aortic aneurysms

Condition	Key differences
IHD	Later onset in women; more non‑obstructive CAD; longer delay in seeking care; PCI favored over CABG in women.
HF	HFpEF is more common in women; men have more RV failure/SCD; women respond better to ARNI/spironolactone; women live longer with HFrEF but with worse QoL; CRT is more beneficial for women; fewer women receive transplants.
HTN	Higher BP in men until menopause, then rises in women.
AS	More common in men
Stroke and carotid disease	Women: More cardioembolic strokes, greater disability, higher stroke risk; Men: More lacunar strokes and carotid stenosis.
Aortic disease	AAA and thoracic aneurysms are more common in men; rupture risk is higher in women.
DM	Higher cardiovascular risk in women.
PAD	Women have atypical/no leg symptoms, receive fewer therapies, and present with more advanced disease.

## Conclusions

Sex-specific differences in CVD extend across the spectrum of IHD, HF, HTN, valvular disorders, cerebrovascular disease, aortic pathology, diabetes, and peripheral arterial disease. These differences are not only biological but also reflect disparities in recognition, diagnosis, treatment, and outcomes. Women often experience atypical symptom presentation, diagnostic delays, and lower utilization of guideline-directed therapies, all of which contribute to worse prognoses despite lower overall disease prevalence in some conditions.

Ultimately, closing the gap in cardiovascular outcomes requires targeted actions supported by existing evidence. Routine sex-stratified reporting of clinical trial outcomes and mandated sex/gender enrollment targets would improve the evidence base and reduce persistent underrepresentation of women. Incorporating sex-specific diagnostic thresholds, risk algorithms, and guideline triggers, such as tailored symptom criteria, biomarker cutoffs, and anatomical thresholds, would directly enhance clinical accuracy. Clinician education on atypical and non-chest-pain presentations in women should be formalized in training and continuing education. These measures provide a more actionable framework for advancing equitable cardiovascular care.

Continued progress in sex-specific cardiovascular medicine requires coordinated efforts across foundational science, clinical research, and health-system implementation. Mechanistic priorities include genomics, proteomics, metabolomics, and hormonal pathway studies to clarify biological drivers of sex differences in disease susceptibility, progression, and therapeutic response. Clinical trial innovation is needed through adaptive designs, sex-stratified randomization, mandatory prespecified sex-based analyses, and adequate statistical power to detect sex-treatment interactions. Implementation science should address how to effectively integrate sex-specific diagnostic thresholds, symptom profiles, guideline triggers, and treatment algorithms into everyday clinical workflows, including evaluating which strategies best overcome persistent diagnostic delays in women. Health equity research must define and mitigate structural determinants of sex disparities, such as under-screening, delayed referral, and inequitable access to advanced therapies. Finally, patient-reported outcome measures require rigorous validation across sexes to ensure accurate assessment of symptom burden, quality of life, and treatment benefit in both women and men.
